# Interdisciplinary Approaches of Transcranial Magnetic Stimulation Applied to a Respiratory Neuronal Circuitry Model

**DOI:** 10.1371/journal.pone.0113251

**Published:** 2014-11-18

**Authors:** Stéphane Vinit, Emilie Keomani, Thérèse B. Deramaudt, Victoria M. Spruance, Tatiana Bezdudnaya, Michael A. Lane, Marcel Bonay, Michel Petitjean

**Affiliations:** 1 Université de Versailles Saint-Quentin-en-Yvelines, Unité End:icap, UFR des Sciences de la Santé – Simone Veil, Montigny-le-Bretonneux, France; 2 LIA-BAHN (Laboratoire International Associé – Biologie Appliquée Handicap Neuromusculaire), CSM (Centre Scientifique de Monaco), Monaco, Monaco; 3 Drexel University College of Medicine, Department of Neurobiology and Anatomy, Philadelphia, Pennsylvania, United States of America; 4 Service de Physiologie-Explorations Fonctionnelles, Hôpital Ambroise Paré, Assistance Publique-Hôpitaux de Paris (AP-HP), Groupe Hospitalier Paris Ile-de-France Ouest, Boulogne-Billancourt, France; University of Kentucky, United States of America

## Abstract

Respiratory related diseases associated with the neuronal control of breathing represent life-threatening issues and to date, no effective therapeutics are available to enhance the impaired function. The aim of this study was to determine whether a preclinical respiratory model could be used for further studies to develop a non-invasive therapeutic tool applied to rat diaphragmatic neuronal circuitry. Transcranial magnetic stimulation (TMS) was performed on adult male Sprague-Dawley rats using a human figure-of-eight coil. The largest diaphragmatic motor evoked potentials (MEPdia) were recorded when the center of the coil was positioned 6 mm caudal from Bregma, involving a stimulation of respiratory supraspinal pathways. Magnetic shielding of the coil with mu metal reduced magnetic field intensities and improved focality with increased motor threshold and lower amplitude recruitment curve. Moreover, transynaptic neuroanatomical tracing with pseudorabies virus (applied to the diaphragm) suggest that connections exist between the motor cortex, the periaqueductal grey cell regions, several brainstem neurons and spinal phrenic motoneurons (distributed in the C3-4 spinal cord). These results reveal the anatomical substrate through which supraspinal stimulation can convey descending action potential volleys to the spinal motoneurons (directly or indirectly). We conclude that MEPdia following a single pulse of TMS can be successfully recorded in the rat and may be used in the assessment of respiratory supraspinal plasticity. Supraspinal non-invasive stimulations aimed to neuromodulate respiratory circuitry will enable new avenues of research into neuroplasticity and the development of therapies for respiratory dysfunction associated with neural injury and disease (e.g. spinal cord injury, amyotrophic lateral sclerosis).

## Introduction

There is a wide spread appreciation for developing new and powerful non-invasive strategies, particularly enhancing neural activity and neuroplasticity with the therapeutic potential of transcranial magnetic stimulation. While numerous studies have reported various effects of repetitive transcranial magnetic stimulation (rTMS) on different rat cortical and brainstem structures by increasing neuroplasticity involved molecules [1], [2], the effect of rTMS on respiratory function and respiratory neuroplasticity remains unknown. Interdisciplinary synergies (neuroanatomy and electrophysiology principally) have been the first crucial step to address this gap in knowledge.

Cortical influence over respiratory neuronal control has been demonstrated in several animal preparations [3] and clinically in humans [4]–[6], by electrically stimulating primary motor cortex and recording motor evoked responses with diaphragmatic electromyograms. Although the precise anatomical and functional relationship between cortical and bulbar neurons remains unclear, there is some physiological evidence for a descending corticospinal pathway innervating the phrenic motoneuron pool in cat [7] and rat [8]. Transcranial magnetic stimulation (TMS) in humans has proven to be effective in the study of diaphragmatic motor evoked potential (MEP) [9]–[13]. This provides an easy, non-invasive and painless clinical tool for studying respiratory supraspinal pathway excitability and plasticity in response to neuromodulation techniques in humans and animals [14], [15]. Recent clinical studies in healthy patients [16]–[18] have demonstrated that repetitive transcranial magnetic stimulation (rTMS) and transcranial direct current stimulation (tDCS) could increase or decrease diaphragmatic MEP amplitude respectively in response to a TMS single pulse. In case of respiratory neuronal disordered breathing patients such as partial tetraplegia or amyotrophic lateral sclerosis, the ability of supraspinal neuromodulation techniques, such as rTMS, to potentiate diaphragmatic output could lead to respiratory functional recovery. However, clinical studies in respiratory deficiency patients are extremely limited and having a viable, reliable and quantifiable preclinical respiratory model is dramatically needed to further understand the consequences of supraspinal neuromodulation as therapeutics. Based on the results from these human studies, the aim of the present work was 1) to demonstrate the suitability of these stimulation techniques to the supraspinal respiratory pathways of adult rats and 2) to determine whether TMS represents a therapeutic strategy for enhancing diaphragmatic motor activity and neuroplasticity in preclinical models aimed to mimic various human respiratory insufficiencies.

## Materials and Methods

### Ethics statement

All experiments reported in this manuscript conformed to policies laid out by the National Institutes of Health (USA) in the Guide for the Care and Use of Laboratory Animals. The majority of these experiments were performed on 2 month-old male Sprague–Dawley rats (Janvier, France). The animals were dual-housed in individually ventilated cages in a state-of-the-art animal care facility (2CARE animal facility, accreditation A78-322-3, France), with access to food and water *ad libitum* with a 12 h light/dark cycle. These experiments were approved by the Ethics committee of the RBUCE-UP chair of Excellence (University of Paris-Sud, grant agreement No. 246556) and the University of Versailles Saint-Quentin-en-Yvelines. Neuroanatomical tracing was performed at Drexel University, College of Medicine (Philadelphia, USA) using male and female adult Sprague-Dawley rats (purchased from Harlan Laboratories). Tracing studies were also conducted with Institutional Animal Care and Use Committee (Drexel University) approval. This manuscript has been prepared in accordance with ARRIVE guidelines.

### Transcranial magnetic stimulation (TMS)

TMS was performed using a magnetic stimulator MAGPRO X100 (Magventure, Farum, Denmark) connected to a figure-of-eight coil (CB60; dimensions: 165×85×20 mm) delivering a unique biphasic pulse (380 µs in duration) with an intensity of the stimulus expressed as a percentage of maximum output of the stimulator (% MO), from 60 to 100% MO in this study. Magnetic shielding was established by inserting a 2 mm thick plate of Mu-METAL (Magnetic Shield Corp, Bensenville, IL, USA) designed by MecaMagnetic (Amilly, France) (85 mm×165 mm), with a 30×30 mm centered open window, between the coil and the animals scalp (group labeled “Shielded” in the figure). MuMETAL is an alloy patented and distributed worldwide by Magnetic Shield Corp (Bensenville, IL, USA). MuMETAL is made with Nickel (80%), Iron (15%) and Molybdene (5%) (MECAMAGNETIC, Amilly, France). The total size corresponds to the coil dimensions. The “Unshielded” group throughout the manuscript refers to the use of the coil without magnetic shielding.

### Magnetic field quantification

Mapping of the magnetic field intensities was done to evaluate the influence of magnetic field shape over the coil relative to the rat central nervous system structures, and the effect of a magnetic shielding with a centered open window. Single magnetic pulses were recorded using a solenoid (Magprobe, Magventure, Farum, Denmark). The solenoid is a 2.5 cm diameter copper coil converting a magnetic field into a measurable electrical current (in Volts). The solenoid was placed as close as possible to the CB60 coil (570 different points of recording, 30×19 matrix) in all three dimensional directions of the magnetic field (x, y and z axes). The induced electrical current for each direction (x, y and z) was recorded by using the Powerlab device and the LabChart 7 Pro software (AD Instrument). The whole magnetic field recorded with the solenoid was calculated using the following formula: √(x^2^+y^2^+z^2^). Each dimension of the electrical field induced in the solenoid was reconstructed in 3D with a 2D Z-axis projection (Sigmaplot 12.5 software).

### Electrophysiological recordings

A total of 20 animals were used for terminal electrophysiological assessment, randomly divided into those that received a single pulse of TMS with magnetic shielding on the stimulating coil (“Shielded, n = 9) and those that have no shield on it (“Unshielded”, n = 11). Each animal received about 100 TMS single pulses throughout the different experimental conditions. Interpulse duration was always above 10 s to avoid low frequency repetitive TMS like effects known to induce neuroplasticity and dramatically reduce motor excitability [14], [15]. As described previously [19], anesthesia was induced using isoflurane (100% O_2_ balanced). A 25 G catheter was placed in the tail vein, then the rats were tracheotomized and pump ventilated (Rodent Ventilator, model 683; Harvard Apparatus, South Natick, MA, USA). The ventilation rate was adjusted to maintain the animal below its central apneic threshold throughout the experiment. EtCO_2_ was monitored using an infrared capnograph (Viamed, VM-2500-M). Anesthesia was maintained using isoflurane (3.5% in 21% O_2_, continuously monitored by an oxygen sensor (Viamed, AX300). Animals were placed on a heating pad to maintain a constant body temperature and rectal temperature was monitored throughout the experiment. A catheter was inserted in the right femoral artery to measure arterial pressure. Arterial and tracheal pressures were monitored continuously with transducers connected to a bridge amplifier (AD Instruments). Isoflurane anesthesia was then slowly converted to urethane anesthesia (1.8 g/kg, i.v.; Sigma-Aldrich). A single dose of urethane is sufficient to keep the animal deeply anesthetized throughout the experiment. The depth of anesthesia was confirmed by the absence of any response to toe pinch. A laparotomy was performed, and the liver was gently moved caudally to access the diaphragm. Gauze soaked with warm buffered saline was placed on the liver to prevent dehydration.

A custom-made silver bipolar electrode was placed on the right mid-costal part of the diaphragm. Diaphragm EMG and motor evoked potential induced by a single pulse of TMS (MEPdia) were amplified (gain, 1 k; A-M Systems, Everett, WA, USA) and band pass-filtered (100 Hz to 10 kHz). The signal was digitized with an 8-channel Powerlab data acquisition device (Acquisition rate: 100 k/s; AD Instruments) connected to a computer. The data was recorded and analyzed using LabChart 7 Pro software (AD Instruments). The head of the animal was placed on a non-magnetic custom-made stereotaxic apparatus which allowed moving the head of the animal from the center of the figure-of-eight coil along rostro-caudal axis and rotational positions in line with previous publications [9], [20]–[24]. At the end of the study, a bilateral vagotomy followed by a paralytic agent (gallamine trietiodide, 1 mg, Sigma, n = 8, 4 from each group) or a complete transection of the spinal cord at the C2 level (n = 8, 4 from each group) were performed on all animals. At the end of the experiment (around 3 hours of anesthesia), the animal is euthanized with an i.v. overdose of urethane.

### Neuroanatomical Tracing

A total of 8 adult Sprague Dawley rats (n = 4 female; n = 4 male) were used for transneuronal retrograde tracing studies to determine the distribution of neurons throughout the brain and brainstem that are associated with the phrenic circuitry. While female rats have been used for previous similar transneuronal tracing studies [25], [26], the present study used males also to be consistent with those animals used for TMS and ensure there were no gender-specific differences in underlying circuitry. As previously described [25], animals were surgical anesthetized and received a laparotomy to expose the peritoneal surface of the diaphragm. The transneuronal tracer pseudorabies virus (PRV614, 2.0×10^8^ pfu) was topically applied to the entire surface of the left hemi-diaphragm. Musculature and skin were then sutured and closed with wound clips, respectively. Animals were given lactated Ringer’s solution (5 ml subcutaneous) to prevent dehydration, Yohimbine (1.2 mg/kg subcutaneous) as a xylazine reversal agent, and buprenorphine (0.05 mg/kg subcutaneous) for pain relief.

### Histology and Microscopy

Seventy two hours following PRV-delivery, animals used for neuroanatomical tracing were euthanized with either Beuthanasia (n = 4; 9∶1, sodium pentobarbitone to phenytoin solution; 0.45 ml i.p.) or Euthasol (n = 4; 0.5 ml i.p.). Tissues were then intracardially perfuse-fixed with paraformaldehyde (4% w/v in 0.1 M phosphate buffered saline (PBS), pH ∼7.4). Spinal cord and brain tissue was dissected and post-fixed by immersion in paraformaldehyde (4%). Brain, brainstem and cervical spinal cord tissues were cryoprotected (sucrose, 30% w/v in 0.1 M PBS) and frozen. Transverse sections (Cryostat, 40 micron thick) were made throughout the entire brain, brainstem and upper cervical spinal cord. Every section was immunolabeled with primary antibodies for PRV (Rabbit anti-PRV (Rb134) - raised against whole, purified PRV particles that were acetone inactivated; provided by Dr. Lynn Enquist, Princeton University, as a service of the National Center for Experimental Neuroanatomy with Neurotropic Viruses: NCRR P40 RRO118604). Prior to immunohistochemical methods, floating sections were washed in PBS (0.1 M (pH = 7.4), 3×5 min), blocked against endogenous peroxidase activity (30% methanol, 0.6% hydrogen peroxide in 0.1 M PBS, incubated for 1 h), re-washed in PBS and blocked against non-specific protein labeling (10% serum in 0.1 M PBS with 0.03% Triton-X, incubated for 1 h). Tissue was then incubated in Rb134 at 4°C overnight. The following day, sections were washed (3×PBS), incubated in secondary antibodies (biotinylated donkey anti-rabbit, Jackson Immunocytochemicals; 1∶200) for 2 hours at room temperature, and washed again (3×PBS). Finally, sections were incubated in a Vectastain ABC solution (2 hours at room temperature), washed (3×PBS) and processed for DAB reactivity. Labeled sections were washed, slide mounted, counterstained with cresyl violet and coverslipped. Sections were examined using a Zeiss AxioImager M2 with motorized stage and images captured using an AxioCam HRc (high resolution, color digital camera) and Zen Pro 2012 software on a PC computer. For images taken with a 40x objective, Z-stacks were created (10–13 images, ∼1.0 micrometer apart) and processed for extended depth of focus images.

### Data processing

A minimal average of 5 MEPdia for each condition was calculated with LabChart Pro software (AD Instruments). The peak-to-peak amplitude of the first negative wave (N1) of each averaged MEPdia were measured. MEP latency was defined as the first electrical (positive or negative) deviation following the magnetic pulse artefact. Given that MEP onset latency can be difficult to determine, the first negative peak latency was also used (as has been described previously in rodent TMS studies [27]).

Normality of data distribution was assessed using a Kolmogorov-Smirnoff test and log transformation was performed if necessary. Two way ANOVA with Bonferonni correction for multiple comparisons were performed between animal’s groups followed by post-hoc Student t-test. A Student paired t-test was performed to demonstrate the effect of magnetic shielding. All the data are presented as mean ± one SEM. A test was considered significant if p<0.05.

## Results

### Effect of magnetic shield on the magnetic field generated by the TMS coil

**Figure 1:**
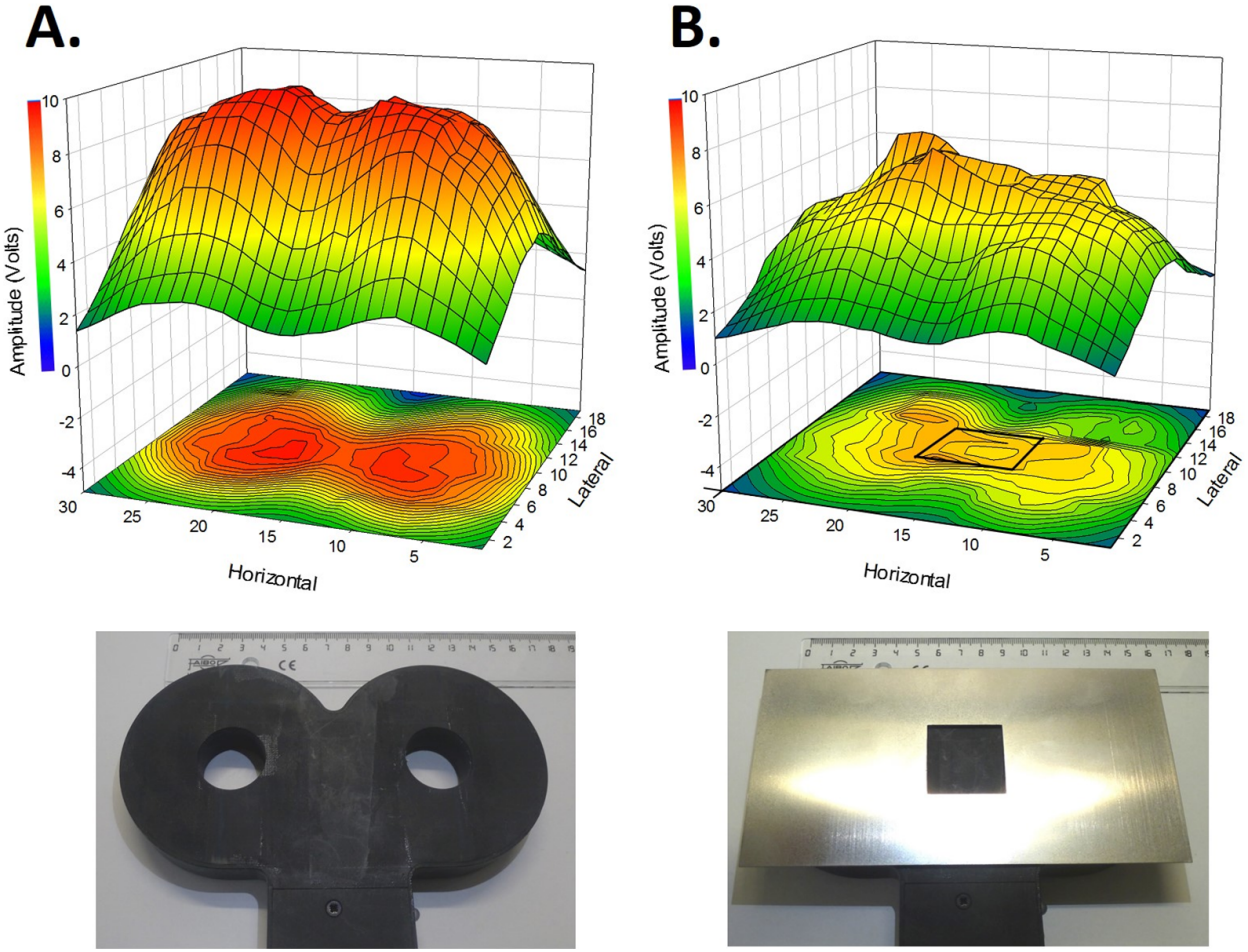
Effect of magnetic shield on the induced magnetic field intensity from the TMS coil. A) 3D representation and its 2D projection (bottom of the graph) of the whole magnetic field intensity (represented as intensity colors) recorded in volts with the solenoid from the “Unshielded” TMS coil. Picture at the bottom represents the coil. B) 3D representation and its 2D projection (bottom of the graph) of the whole magnetic field intensity (represented as intensity colors) recorded with the shield on the TMS coil (Noted as “Shielded”). Picture at the bottom represents the coil with the shield on it with the 30×30 mm window. Note that black square in the 2D projection representing the 3×3 cm window. The intensity is statistically reduced by 32% (p<0.001) compared to the one obtained in the “Unshielded” coil, and better concentrated where the rat head will be placed on (Middle of the window). Horizontal and Lateral scale represent the side of the coil, which each measured point represents half centimeter.

The entire recorded magnetic field is located in the center of the coil without the magnetic shield, with two hot spots located in the middle of the two holes of the figure-of-eight (represented in red, Figure 1A). The magnetic field is statistically reduced by 32% with shielding (average “Unshielded” = 5.76±0.1 V, average “Shielded” = 3.89±0.06 V, p<0.001, paired t-test; Figure 1A and 1B) and focused in the center of the 30×30 mm window (Figure 1B). All the 3 dimensional x, y and z of the magnetic field are statistically reduced in presence of the magnetic shield (average “Unshielded” in x = 3±0.09 V, average “Shielded” in x = 2.03±0.06 V, reduction of 32%, p<0.001, paired t-test; average “Unshielded” in y = 2.74±0.07 V, average “Shielded” in y = 1.78±0.04 V, reduction of 35%, p<0.001, paired t-test; average “Unshielded” in z = 2.75±0.11 V, average “Shielded” in z = 2.07±0.07 V, reduction of 24%, p<0.001, paired t-test).

### Physiological effect of a single magnetic pulse on the diaphragmatic motor evoked response (MEPdia)

**Figure 2:**
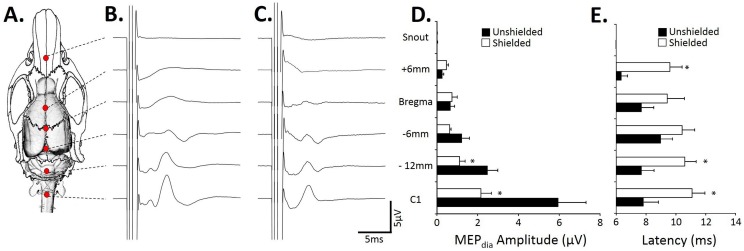
Antero-posterior stimulation with a single TMS pulse on the amplitude and latency of the observed MEPdia. A) Representation of the different stimulated sites on a rat brain skull with a schematic representation of the rat brain and upper spinal cord. B) Representative MEPdia induced with a single stimulation using the “Unshielded” coil from different rostro-caudal stimulation sites. C) Representative MEPdia induced with a single stimulation using the “Shielded” coil from different rostro-caudal stimulation sites. D) Histogram of the MEPdia amplitudes (in µV) from the different rostro-caudal stimulated position with “Shielded” (white) and “Unshielded” (black) coil in all animals. E) Histogram of the MEPdia latencies (in ms) from the different rostro-caudal stimulated positions with “Shielded” (white) and “Unshielded” (black) coil in all animals. *: p<0.05.

No statistical differences were observed in physiological parameters (body weight, body temperature, EtCO_2_, blood pressure) between treatment groups (“Unshielded” vs. “Shielded”; Table 1). A single pulse of magnetic stimulation at different locations of the brain and spinal cord (Figure 2A) induced different MEPdia depending on the origin of the stimulus. A stimulation (100% MO for the “Shielded” group; 85% MO for the “Unshielded” group, representing 140% of resting motor threshold (MT) value in each condition) applied at the C1 spinal cord, 12 and 6 mm caudal to Bregma (−12 and −6), at Bregma, and 6 mm rostral to Bregma (+6) locations, induced an observable MEPdia whereas a stimulation of the snout did not induce a detectable MEPdia in either group (Figure 2B and 2C).

**Table 1:** Physiological parameters.

	Body weight (g)		Temperature (°C)		EtCO_2_ (mmHg)		MAP Start (mmHg)		MAP End (mmHg)	
Treatment Group	Mean	SEM	Mean	SEM	Mean	SEM	Mean	SEM	Mean	SEM
No permalloy	339	16	37.3	0.2	38.8	0.8	108	4	111	5
3×3 cm permalloy	327	21	36.9	0.1	38	1.8	90	5	93	5

The magnetic shield does not affect the presence/absence of the MEPdia. However, it statistically reduced the amplitude of MEPdia for the stimulation at −12 mm caudal to Bregma (“Unshielded”: 2.49±0.48 µV compared to “Shielded”: 1.11±0.26 µV, p = 0.04; Figure 2D) and for the stimulation of the upper cervical spinal cord (C1; “Unshielded”: 5.95±1.34 µV compared to “Shielded”: 2.15±0.51 µV, p = 0.045; Figure 2D). No statistical differences were observed on the MEPdia amplitude for the stimulation at −6 mm from Bregma (“Unshielded”: 1.23±0.36 µV compared to “Shielded”: 0.62±0.06 µV, p = 0.751; Figure 2D), at Bregma (“Unshielded”: 0.67±0.19 µV compared to “Shielded”: 0.74±0.24 µV, p = 0.597; Figure 2D) and +6 mm rostral to Bregma (“Unshielded”: 0.27±0.05 µV compared to “Shielded”: 0.47±0.09 µV, p = 0.102; Figure 2D).

The magnetic shield significantly increased the latency of the recorded MEPdia for the stimulation at C1 location (“Unshielded”: 7.84±0.98 ms compared to “Shielded”: 11.07±0.86 ms, p = 0.009; Figure 2E), −12 mm to Bregma (“Unshielded”: 7.71±0.81 ms compared to “Shielded”: 10.58±0.77 ms, p = 0.031; Figure 2E) and +6 mm to Bregma (“Unshielded”: 6.36±0.39 ms compared to “Shielded”: 9.59±0.82 ms, p = 0.003; Figure 2E). No significant differences in the latency of the MEPdia are observed for the stimulation at Bregma location (“Unshielded”: 7.71±0.79 ms compared to “Shielded”: 9.42±1.14 ms, p = 0.161; Figure 2E) and −6 mm to Bregma (“Unshielded”: 9±0.77 ms compared to “Shielded”: 10.41±0.83 ms, p = 0.252; Figure 2E). However, no difference in MEPdia latency was observed between groups “Unshielded” and “Shielded” at the different locations of the stimuli (p>0.05; Figure 2E).

**Figure 3:**
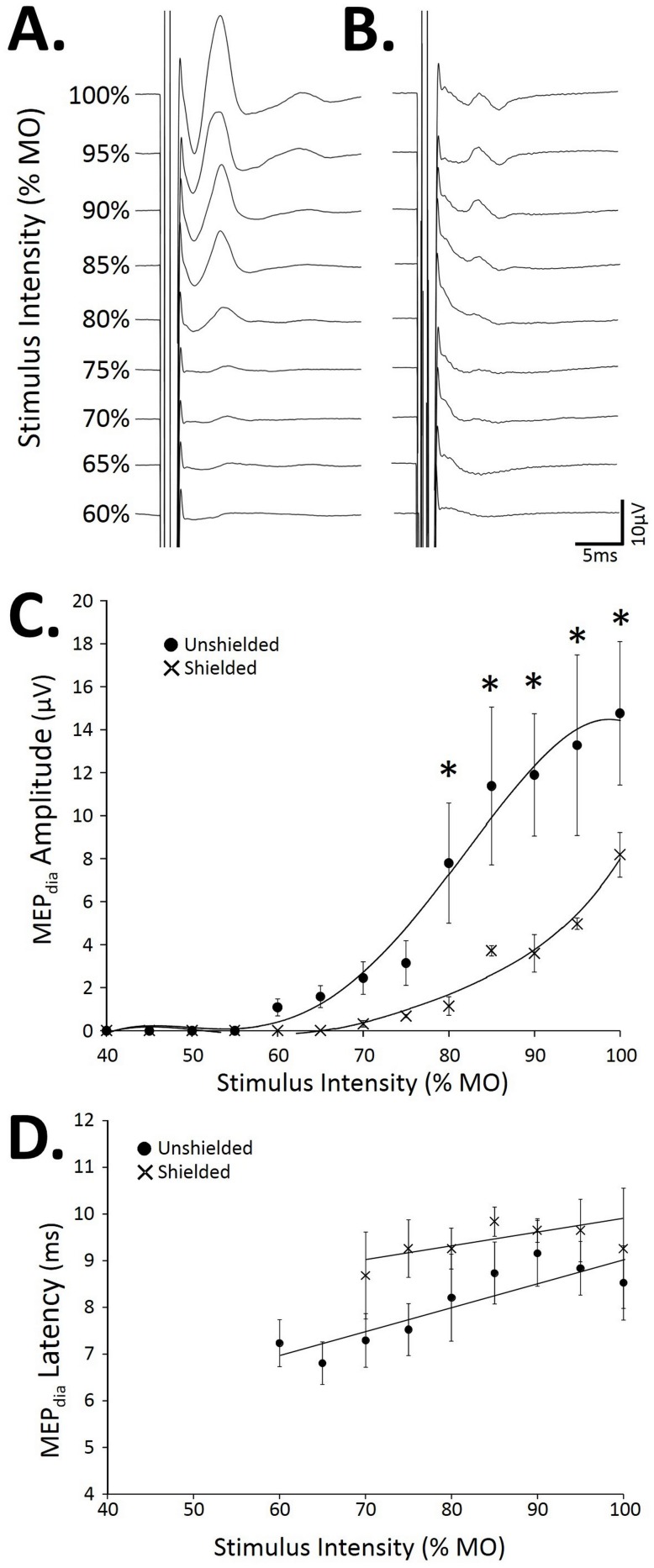
Effect of the stimulus intensity on the MEPdia following a single TMS at −6 mm from Bregma. A) Representative MEPdia obtained with different stimulus intensities (60 to 100% MO) using the “Unshielded” coil. B) Representative MEPdia obtained with different stimulus intensities (60 to 100% MO) using the “Shielded” coil. C) Correlation between MEPdia amplitude (in µV) and stimulus intensity (% MO) for the animals stimulated in “Unshielded” (black circles) or “Shielded” (black x) coil. D) Correlation between MEPdia latency (in ms) and stimulus intensity (% MO) for the animals stimulated with the “Unshielded” (black circles) or “Shielded” (black x) coil. MO: Maximal output; *: p<0.05.

The amplitude of the MEPdia was dependent on the stimulus intensity of the magnetic stimulator (expressed as % of maximum output (MO)). The MEPdia starts to be recorded at a resting motor threshold (MT), namely the stimulus intensity of around 70% MO in both groups (“Unshielded” Figure 3A; “Shielded” Figure 3B) at −6 mm from Bregma’s stimulation site. The amplitude of the observed MEPdia increases with the stimulus intensity in both “Unshielded” and “Shielded” groups (Figure 3A and 3B respectively). Quantitatively, the MEPdia amplitudes for the “Unshielded” group (Figure 3C, black circles) follow a S-shape curve, with a plateau at a stimulus intensity of 85% MO, whereas, for the “Shielded” group (black x), a plateau is not yet reached at the stimulus of 100% MO (MEPdia amplitude at 95% MO = 4.9±0.2 µV compared to 100% MO = 8.1±1.0 µV, p<0.05). A statistical difference in MEPdia amplitudes was observed between groups, starting from 80% MO up to 100% MO (p<0.05). The stimulus intensities do not have any statistical effects (p>0.05) on the MEPdia latency in each group or between groups (Figure 3D) at −6 mm from Bregma.

**Figure 4:**
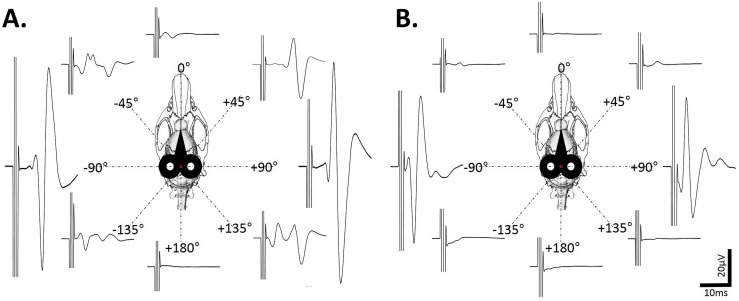
Effect of the coil rotation on the MEPdia. A) Representative MEPdia recorded after a single TMS pulse with the “Unshielded” coil at different orientation degrees (from 0° to 360°, 45° steps) of the coil (black coil represented on the top at −6 mm from Bregma in black). B) Representative MEPdia recorded after a single TMS pulse with “Shielded” coil at different orientation degrees (from 0° to 360°, 45° steps).

Moreover, the orientation of the coil also plays a role in the amplitude, shape and latency of the MEPdia recordings after occipital cortex stimulation (Figure 4). When the coil is at 0°, the figure-of-eight is perpendicular to the rostrocaudal axis of the animals head (See figure 4). The amplitude of the MEPdia in the “Unshielded” group varies with the coil orientation, with a maximum amplitude at +90° and −90° (Figure 4A, the figure-of-eight is in-line with the rostrocaudal axis). This correlates with the shape of the magnetic field (Figure 1A) where the maximal field intensity (in red) is represented in both holes of the eight-shape coil. A similar tendency is also observed in the “Shielded” group with an evident reduction in MEPdia amplitude (Figure 4B).

### Distribution of neurons associated with the phrenic circuitry in the rat model

Transynaptic PRV tracing of spinal and supraspinal neurons associated with the diaphragm on one side provided an insight into those cells that can be stimulated by TMS. The number of labelled cells were not quantified in these tracing experiments as they were intended only to provide a map of the distribution of labelled cells.

**Figure 5:**
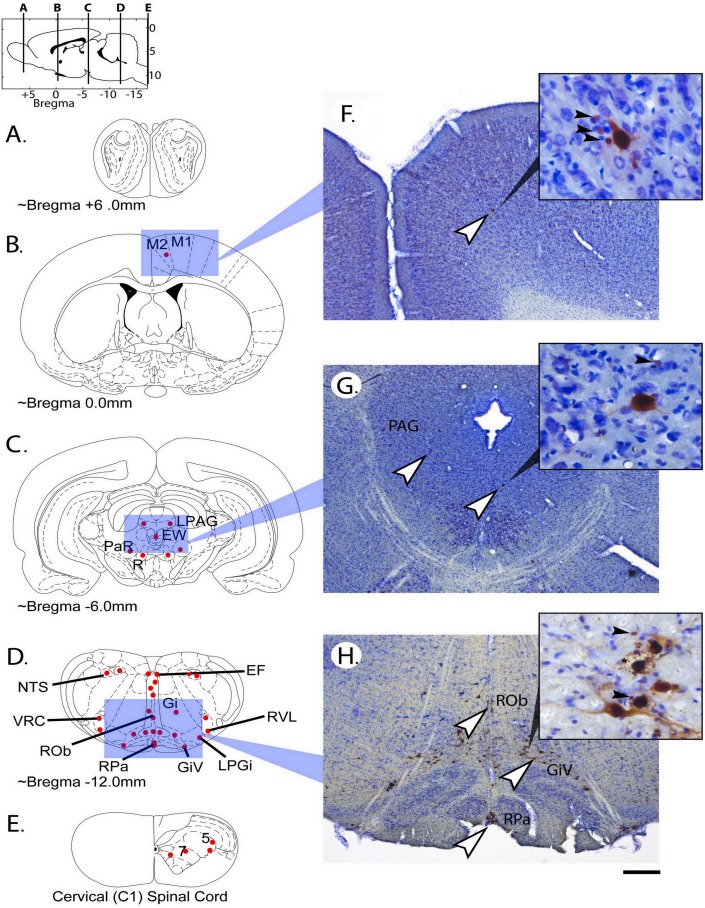
Schematic diagrams (modified from [43]) representing cross sections of the brain (A–C), brainstem (D) and spinal cord (E), and corresponding images of tissue that has been immunolabeled for the presence of PRV (brown; white arrowheads) and counterstained with cresyl violet (F–G). Inset show examples of PRV-labeled neurons at higher magnification. Schematic diagrams are representative of the stereotaxic positions that the TMS coil was used in the present work. Each of these positions relative to Bregma is also indicated on the sagittal diagram above. The qualitative distribution of PRV labelled neurons is indicated by red dots. PRV-positive neurons were detected in the cortex, the midbrain, the medulla, and spinal cord 72 hours following delivery of PRV to the diaphragm. Only few PRV-positive neurons were detected within the motor cortex at this post-PRV time point (primary (M1) and secondary (M2) motor cortex). Approximately 6 mm caudal to bregma, several PRV-positive cells were seen within midbrain in the periaqueductal grey (PAG) and Edinger-Westphal nucleus (EW), and some also in the Red (R) and pararubral nucleus (PaR) nuclei. Several PRV-positive neurons were seen throughout the brainstem 12 mm caudal to Bregma, predominantly in the Raphe (including raphe obscuris (ROb), pallidus (RPa)) and reticular nuclei (gigantocellular (Gi), ventral gigantocellular (GiV), lateral paragigantocellular (LPGi) and rostroventrolateral reticular nucleus (RVL)). Labelling was also seen in two key respiratory centers: the ventral respiratory column (VRC) and the solitary nucleus (nucleus tractus solitarius; NTS) (which comprises the ‘dorsal respiratory group’). Finally some labelling was seen within the epifascicular nucleus. PRV labelled spinal interneurons were observed bilaterally in the C1, primarily in the intermediate grey matter (laminae 7 and 10), but fewer neurons seen in laminae 1, 5, and 8. Note the differences in morphology of PRV-labeled neurons shown in the insets (e.g. late-stage neuronal infection results in cell death (*), additional description in the text) and the presence of some smaller PRV-positive cells (black arrowheads; likely glia). Scale bar is 200 (F–H) and 40 micrometres (insets).

PRV-positive interneurons in the upper cervical (C1) spinal cord were primarily detected bilaterally in laminae 7 and 10, with fewer cells seen also seen in laminae 1, 5 and 8 (see Figure 5). Within the brainstem (Figure 5D, 5H), PRV-labeled neurons were observed in the following regions: serotonergic nuclei (raphe obscuris (ROb); raphe pallidus (RPa); group B5 serotonergic cells), reticular nuclei (gigantocellular (Gi); ventral gigantocellular (GiV); lateral paragigantocellular (LPGi); rostroventrolateral (RVL), the epifascicular nucleus (EF), nucleus of the solitary tract (or nucleus tractus solitarius (NTS) which forms the ‘dorsal respiratory group’), and the ventral respiratory column (VRC; comprised of the rostral and caudal ventral respiratory groups, the pre-Bötzinger and Bötzinger complexes, the retrotrapezoid nucleus and the parafacial respiratory group). Examining tissue more rostrally (∼9 mm caudal of Bregma) also revealed labelling in the periaqueductal grey matter (lateral (LPAG), ventrolateral and dorsomedial, tegmental nuclei, the dorsal aspect of the subcoeruleus and the Kölliker-Fuse nucleus. Around 6 mm caudal of Bregma (Figure 5C, 5G), PRV-positive neurons were seen within the periaqueductal grey matter, the rubral and pararubral nuclei (or Red nuclei, PaR and R) and the Edinger-Westphal nucleus. Another millimetre more rostrally labeled neurons were seen in the lateral hypothalamic area (LH), the reticular aspect of the substantia nigra (SNR), pre-commissural nucleus (PrC) and the ventral part of zona incerta (ZIV). At approximately the level of Bregma, PRV-labeled neurons were seen in the periventricular and paraventricular and hypothalamic nuclei, and the primary (M1) and secondary (M2) motor cortex (Figure 5B). No PRV-positive neurons were detected rostral to those seen in the motor cortex (∼1.5 to 0.0 mm rostral of Bregma).

Early neuronal PRV infection is usually indicated by PRV within the nucleus and cytoplasm, with limited dendritic labelling (Figure 5, inset in G). During later stages of infection PRV is detectable through neuronal dendrites and may be seen in surrounding glia that take up viral particles at synapses (Figure 5, inset in F and H). Final stages of PRV-infection are marked by neuronal pathology associated with cell death, as the cell is compromised by long-term infection (Figure 5, inset in H).

### Specificity of the recorded diaphragmatic motor evoked potential

**Figure 6:**
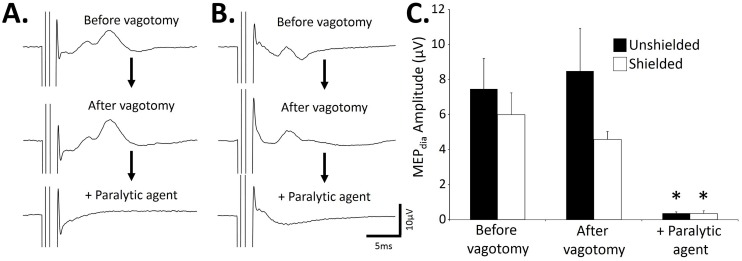
Effect of vagotomy and muscular paralysis on the MEPdia induced by a single TMS pulse at −6 mm from Bregma. A) Representative MEPdia before vagotomy, after vagotomy and injection of a paralytic agent (gallamine trietiodide) obtained with the “Unshielded” coil. B) Representative MEPdia before vagotomy, after vagotomy and injection of a paralytic agent (gallamine trietiodide) obtained with the “Shielded” coil. Note that the vagotomy does not affect the MEPdia, and the paralytic agent abolish the MEPdia in both conditions in A and B. C) Histogram of the MEPdia amplitude before vagotomy, after vagotomy and following the injection of paralytic agent in the “Unshielded” (black histogram) and “Shielded” (White histogram) on the TMS coil. *: p<0.001.

**Figure 7:**
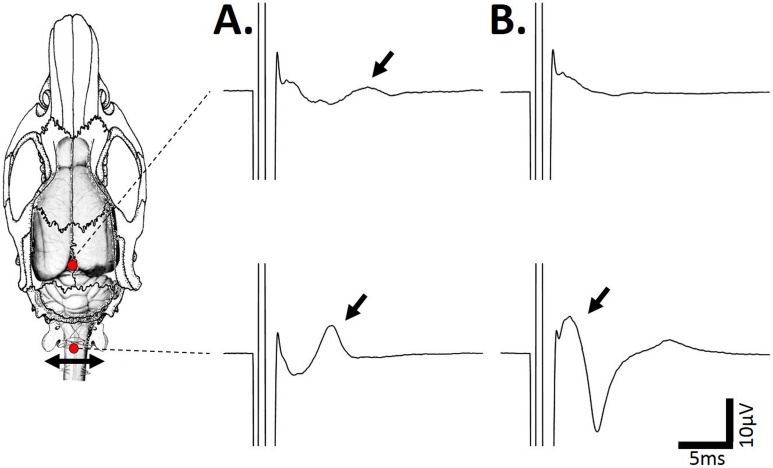
Effect of a total transection of the spinal cord at the C2 level on the MEPdia upon stimulation at −6 mm from Bregma and at the C1 spinal cord. A) Representatives MEPdia (black arrows) obtained after a single TMS pulse at −6 mm from Bregma (top trace) and at C1 spinal cord site (bottom trace) in the “Unshielded” coil group before complete C2 spinal cord transection. B) Representatives MEPdia (black arrows) obtained after a single TMS pulse at −6 mm from Bregma (top trace) and at C1 spinal cord site (bottom trace) in the “Unshielded” coil group after the complete C2 spinal cord transection. Note the absence of MEPdia when the stimulation is applied at −6 mm from Bregma, and the presence of an enhanced MEPdia when the C1 spinal cord is stimulated.

A single magnetic stimulation at −6 mm from the Bregma induced an observable MEPdia before vagotomy in both groups (Figure 6A “Unshielded” and 6B “Shielded”). A bilateral vagotomy did not affect the amplitude of the recorded MEPdia in either the “Unshielded” (before vagotomy: 7.4±1.7 µV, after vagotomy: 8.4±2.4 µV, p = 0.25; Figure 6C) or “Shielded” groups (before vagotomy: 5.9±1.2 µV, after vagotomy: 4.5±0.4 µV, p = 0.36; Figure 6C). No difference in latency was observed (data not shown). However, an injection of a paralytic agent (gallamine trietiodide) abolished the MEPdia in both “Unshielded” (after vagotomy: 8.4±2.4 µV, after muscular paralysis: 0.3±0.08 µV, p = 0.018) and “Shielded” (after vagotomy: 4.5±0.4 µV, after muscular paralysis: 0.03±0.1 µV, p = 0.001) groups (Figure 6C). Magnetic stimulations at −6 mm from Bregma (100% MO) and the C1 spinal cord evoked a MEPdia in the “Unshielded” group (Figure 7A). However, a complete transection of the cervical spinal cord at the C2 level abolished the observable MEPdia after stimulation at −6 mm from Bregma. In contrast, the observable MEPdia is enhanced following a C1 spinal cord magnetic stimulation (Figure 7B). Similar results for all the animals are observed in the “Shielded” group (data not shown).

## Discussion

This study represents the first demonstration of diaphragmatic motor response to TMS in adult rat and provides a rationale for the use of TMS in animal studies of respiratory neuroplasticity. While preliminary in nature, these results support the feasibility of using TMS to study respiratory dysfunction associated with neurological injury and disease. In addition, we predict that these experiments will enable new avenues of research into the development of therapeutic strategies to treat respiratory dysfunction.

Previous studies in rat have demonstrated that limb muscle MEPs could be induced by figure-of-eight coil and suggested that pyramidal tract cells were recruited [27]–[30]. Although fore-and hind-limb muscle MEP have been described in awake animals [29], most studies were carried out in anesthetized rats. Most anesthetics are known to reduce or abolish MEP, although propofol [27], [28], ketamine [31], pentobarbital [30] and anesthetics cocktail with urethane [31] have been successfully used in some rat strains (Wistar, Long-Evans). In the present study, urethane anesthesia in Sprague-Dawley rats did not abolish MEPdia evoked by TMS. Moreover, vital physiological signs (e.g. Table 1) were constant all through the experiments and blood pressure was similar to that previously published [19], [32]. Nevertheless, several technical and physiological features have to be discussed regarding i) the use of a magnetic stimulator connected to a large figure-of-eight coil usually used in humans and ii) the specificity those pathways that are stimulated.

Besides testing the feasibility of evoking a diaphragmatic MEP in rat with a conventional magnetic stimulator, we also tested the influence of magnetic shielding designed to focalize or improve the efficiency of the magnetic field over specific brain regions to more selectively depolarize supraspinal cells associated with the phrenic system. The use of magnetic shield has been reported previously in cats [33], suggesting that the focality and the efficiency of the magnetic field was significantly improved while using it, with lower resting motor threshold values and increased MEP amplitude in the flexor carpi radialis. In contrast, the results from the present work using rats with a much smaller brain relative to the coil size show an increase in resting motor threshold with shielding. Physical measurements demonstrate that the focality of magnetic field density is slightly improved by the shield in the middle of the coil surface whereas the intensity is then globally reduced. These results may partly explain why the “Shielded” recruitment curve in figure 4 looks very different from that seen in the “Unshielded” group. Moreover, a recent paper have been shown that it is possible to lateralize the MEP responses in the rat forelimb [30], suggesting that the magnetic field is concentrated enough to stimulate primary motor cortex individually from the two hemispheres.

It is worth noting that the diaphragmatic resting motor threshold values obtained in our experiments are almost in the same range as the ones observed in rat limb muscles [27], [31] as well as in humans in more recent published studies [17], [34]. Moreover, there is an increase of about 10% of resting motor threshold value by adding the magnetic shield, as was reported with the same size shield in cats [33]. By increasing stimulation intensities, there is an obvious recruitment effect, implying that the magnetic field at the center of the coil is high enough to recruit the most excitable supraspinal cells, which are then able to depolarize phrenic motoneurons. However, shielding seems to limit the effect of ectopic stimulation of extra-cortical cell populations as the recruitment curve in animals with shielded TMS displayed a continuous increase up to a plateau of MEPdia, whereas the “Unshielded” recruitment curve exhibits an upward shift as stimulation intensity reaches a threshold value of 80% MO. In addition, stimulation above 80% MO resulted in an increase in SEM amplitude values, suggesting that a first recruitment mode has changed. With this in mind, given the relatively small region of the rat primary motor cortex area devoted to respiration (compared to the muscles of the extremities [35]) shielded TMS is necessary to limit stimulation at the peripheral regions of the coil and the recruitment of extra cortical excitable cells.

Even with shielded TMS, the possibility that neurons ventral to the cortical layers are stimulated cannot be ruled out. For instance, it is likely that neurons within the periaqueducal grey matter (PAG) which are also shown to be associated with phrenic circuitry (transynaptically labelled with PRV), were stimulated in the present study when the coil was positioned over the occipital cortex (∼6 mm caudal to Bregma). For this reason, it was essential to map the distribution of cells throughout the brain and brainstem that are in some way associated with spinal phrenic neurons. Transynaptic tracing was employed to address this for the preliminary needs of the present study, but further work is needed to quantify the number of labelled neurons within each anatomical region, elucidate the connectivity between each nucleus of cells and assess whether cells within each region have excitatory or inhibitory functions. The distribution of cells outlined in the present work is consistent with previous reports of supraspinal neurons involved with phrenic circuitry [36].

Based on the labelling of neurons within the PAG, it is worth noting electrical stimulation of the neurons within this region in rat has been reported to increase breathing frequency and increase the amplitude of respiratory muscle contraction [37]. Consistent with these previous findings, the results from the present work show that TMS in the region of the PAG can alter respiratory output. This therefore supports the rationale for future studies to investigate whether directly stimulating PAG neurons can modulate respiratory function and plasticity, and whether TMS of deep brain stimulation of the PAG could serve as a therapeutic tool to enhance respiratory activity.

The present work also revealed interactions between “Shielded” and “Unshielded” stimulation conditions, and rostro-caudal position of the coil. Without shielding, no MEP was recordable with the coil positioned over the snout even for the highest stimulation intensities. In contrast, stimulation revealed greater recordable MEP at stereotaxic locations more caudally, where PRV-positive neurons were observed within the respiratory motor cortex and periaqueducal grey matter areas. These results are consistent with a previous report describing an excitable region at Bregma (electrically or magnetically evoked MEP [31]). However, a bigger electrical response was recorded when the coil was located over the cerebellum/brainstem regions and the cervical spinal cord. This is to be expected given the density brainstem neurons associated with respiratory control (e.g. the dorsal and ventral respiratory columns), and the transynaptically labelled cells shown in the present tracing studies and reported previously [26], [36], [38]. It should also be noted that cervical neuroforamina root stimulation at high stimulation intensities (almost around 80–90% MO) is possible in animal models [39], and is known to spread out stimulation sites of peripheral motor fibres [40].

### Effects of an acute C2 spinal cord transection

When an acute cervical cord total C2 transection was performed (figure 7), MEPdia could no longer be obtained with a single pulse of TMS at −6 mm from Bregma, confirming that the signal had been relayed to spinal neurons. Nevertheless, following a spinal cord complete transection and magnetic stimulation performed over cervicomedullary region (−12 mm from Bregma), an increased MEPdia amplitude and reduced latency are observed. This increased MEPdia could be due to the removal of inhibitory connections and/or hyperexcitability of the spinal stub due to the release of glutamate following complete injury. Thus C1 stimulation in both unshielded and shielded groups must result in activation of neurons caudal to the C2 transection. These results as well as those from antero-posterior stimulation site shifting indicated the specificity of cortical excitability without any contamination from spinal cord excitable cells when a MEPdia is recorded.

Results concerning the MEP amplitude were confirmed with MEP latency analyses. Data from figure 2, examining the effect of antero-posterior position of the coil, do not show significant change in MEPdia first negative wave (N1) peak latency mean values for the most posterior sites – over cerebellum (−12 mm from Bregma) and cervicomedullary sites (positioned over the C1 spinal cord) – suggesting that closer to the diaphragm, different cell populations could be depolarized and emit action potential volleys with lower propagation velocity. In addition, an antidromic volley could inhibit output from upper respiratory pathways, resulting in the same latency. Thus, it is important to consider the stereotaxic location of stimulation and whether it has the potential to attenuate evoked responses and affect latency related to magnetic shielding. While not significantly different, MEPdia latency mean values were always shorter in unshielded than in shielded conditions. This suggests that shielding would definitively induce a small delay due to the fact that magnetic stimulation would be more effective at motor cortex than supraspinal level. In addition, latency mean values are in the same range as those reported for limb muscles taken either the onset latency [28], [29], [31] or the MEP first negative wave latency (N1) [27] suggesting that evoked potentials originate in the same central nervous system structure rather than at spinal or phrenic nerve locations.

Definitively, our data suggest that the position of the coil over the cortex or deeper brain structures is crucial to record specific diaphragmatic MEP in a rat model, as has been described in humans [9], [11]. MEP amplitude showed a higher response when the coil is centred over specific motor cortex area.

Consistent with previous work [9], the present study also revealed that rotating the orientation of the coil (see Figure 4) resulted in robust differences in MEP. MEP amplitude was increased for −90° as well as +90° positions, which is likely due to the geometry of the magnetic field under the coil, with or without shielding. These two positions correspond to the alignment of the greater axis of the coil and rostro-caudal (antero-posterior) axis of the central nervous system. This also extends the magnetic field over the cervical spinal cord increasing the likelihood of depolarizing phrenic motoneuron axons at neuroforamina portions of the cervical roots. This is not shield dependant, and it is likely that the reduction in the magnetic field is less at the widest part of the coil than in the center. Moreover, the fact that almost no signal was recorded for +180° gives some insight into the influence of magnetic field direction.

Human TMS studies [9], [22] have revealed that the motor cortex is sensitive to magnetic field direction and preferentially recruited for 45° position. This had been interpreted as the orientation of the magnetic field, being perpendicular to primary motor cortex cell orientation in the frontal sulcus [9], [23]. However, the rat cortex is much less complex than human cortex (lissencephalic vs gyrencephalic). Given that stimulation of the rat motor cortex may be affected by coil orientation in a similar manner to that seen in humans, there may be a closer correlation between magnetic field geometry and motor cell intrinsic excitable properties, than with brain surface architecture (as has been suggested in humans).

The major goal of this paper was to demonstrate that recorded MEPdia reflects a response to direct activation of supraspinal respiratory pathways following stimulation of cortical neurons or deeper brain structure stimulation in order to modulate the activity of these pathways to induce a respiratory neuroplasticity. As previously discussed, the correlation between the MEPdia latency and amplitude with the magnitude of the magnetic stimulation output, suggests that the recorded MEP is initiated at the cortical level [41]. However, we cannot rule out that MEPs might be contaminated by stimulation of neurons within other brain and peripheral structures. For example, the size and wideness of the magnetic field induced by a single stimulation could activate respiratory related nerves, such as the vagus nerve, by which one pass through some diaphragmatic afferents. However, despite the fact that the magnetic field is large and powerful enough to depolarize antidromically vagus nerve fibres and could induce an ectopic activation of some diaphragmatic motor units, no changes in MEPdia following a bilateral vagotomy was observed. This confirms the specificity of the magnetic stimulation.

In addition, no MEPdia were recorded following a magnetic stimulation if the animal was pre-treated with a cholinergic blocker (gallamine trietiodide)to induce muscular paralysis. This result reinforces that diaphragmatic motor unit recruitment recorded as a MEPdia is induced by TMS activating supraspinal neurons, and not directly or by local electrical currents induced by the magnetic field depolarized phrenic motor axons [42]. The recorded MEPdia is therefore the consequence of a single TMS pulse which induces an action potential volley from stimulated supraspinal neurons (cortical or deeper brain structures) to phrenic motoneurons innervating the diaphragm. Future studies should employ direct stimulation of brain structures to further elucidate which regions have the greatest influence over respiratory activity and plasticity. Knowledge of these structures could identify targets for developing and testing the potential therapeutic benefits of TMS in order to enhance respiratory corticospinal excitability and/or activate and strengthen pre-existing pathways.

The present work suggests that TMS may be a safe, non-invasive and readily usable strategy for neuromodulation and treatment of respiratory function. The therapeutic effects of TMS can be easily investigated further with either molecular or functional techniques. While these experiments are preliminary and the extent to which TMS applied repeatedly (rTMS) can be used therapeutically needs to be more extensively characterized, there are a wide range of potential therapeutic applications for TMS that can now be explored. Future experiments should investigate whether rTMS could be used to treat respiratory deficits associated with neurological injury and disease.
